# Successful Management of Osimertinib-Induced Heart Failure

**DOI:** 10.3390/medicina58020312

**Published:** 2022-02-18

**Authors:** Atsuko Fukuo, Teruhiko Imamura, Hiroshi Onoda, Koichiro Kinugawa

**Affiliations:** The Second Department of Internal Medicine, University of Toyama, 2630 Sugitani Toyama, Toyama 930-0194, Japan; db5ibefg6r@gmail.com (A.F.); ohiro0203@gmail.com (H.O.); kinugawa-tky@umin.ac.jp (K.K.)

**Keywords:** heart failure, cancer, onco-cardiology

## Abstract

Cancer therapeutics-related cardiac dysfunction is currently of great concern as one of the pivotal therapeutic targets of onco-cardiology. Only a few studies have reported the occurrence of heart failure following the administration of osimertinib, a third-generation epidermal growth factor receptor (EGFR)-tyrosine kinase inhibitor for EGFR mutation-positive advanced non-small cell lung cancer. We report on a 74-year-old woman with osimertinib-induced advanced heart failure with reduced ejection fraction, which was treated by the temporal termination of osimertinib and neurohormonal blocker therapy, as well as heart rate modulation therapy using ivabradine. Despite osimertinib-induced heart failure being relatively rare, aggressive neurohormonal blocker therapy using ivabradine if applicable, as well as the temporal termination of osimertinib, might be a promising therapeutic strategy.

## 1. Introduction

At present, cancer therapeutics-related cardiac dysfunction is a major concern as one of the pivotal therapeutic targets of onco-cardiology [1]. Some of the major antitumor agents that have cardiotoxicity are compounds of the anthracycline family, which directly impair cardiomyocytes and induce irreversible cardiomyopathy in a dose-dependent manner [2]. Anti-HER2 agents are another major type of antitumor agent that exert cardio-toxicity; however, this is reversible at least partially and dose-independent [2].

However, the cardiotoxicity of osimertinib, a newly introduced third-generation epidermal growth factor receptor (EGFR)-tyrosine kinase inhibitor (TKI) [3], remains unclear. Osimertinib has been shown to improve clinical outcomes in T790M-positive non-small cell lung cancer patients who receive other EGFR-TKI therapy, and is now in the frontline setting in EGFR-mutated non-small cell lung cancer [3]. Of note, the therapeutic strategy for osimertinib-induced heart failure remains unestablished. We report a patient with osimertinib-induced heart failure that was successfully managed by the temporal termination of osimertinib and heart rate modulation-supported neurohormonal blocker therapy.

## 2. Case Report

### 2.1. Before Admission

A 74-year-old woman received post-surgical first and second-line chemotherapy following a thoracoscopic left lower lobectomy to treat non-small cell lung cancer seven years ago (EGFR gene-positive and T790 mutation-negative). Two years ago, she was referred to our clinic prior to the administration of the third-line chemotherapy osimertinib to treat recurrent lung cancer (T790 mutation-positive conversion).

**Figure 1:**
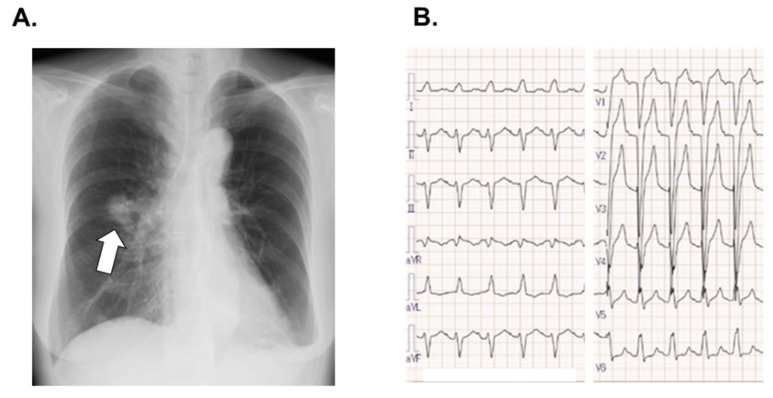
Chest X-ray displaying right lung consolidation (white arrow) and no pulmonary congestion (**A**), and electrocardiogram presenting sinus rhythm and complete left bundle branch block (**B**) obtained at the initial outpatient clinic visit before administration of osimertinib.

Chest X-ray displayed right lung consolidation but no lung congestion (Figure 1A). Electrocardiogram showed normal sinus rhythm with heart rate of 122 bpm, complete left bundle branch block, and QT duration of 351 msec (Figure 1B). Transthoracic echocardiography demonstrated left ventricular end-diastolic diameter of 43 mm and left ventricular ejection fraction of 63%. We initiated oral administration of osimertinib 80 mg. Osimertinib was continued intermittently for two years with monitoring her symptoms and electrocardiograms. QT duration remained around 330 msec.

### 2.2. On Admission

**Figure 2:**
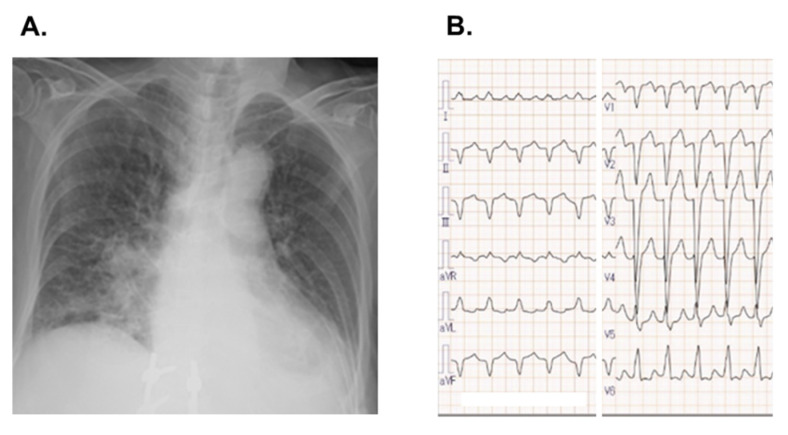
Chest X-ray showing cardiomegaly and bilateral pulmonary congestion (**A**) and electrocardiogram indicating remaining complete left bundle branch block and de novo poor R progression in V1–4 (**B**), obtained on admission following the initiation of osimertinib.

From one month, bilateral leg edema and dyspnea on exertion had emerged. She was admitted to our institute complaining of worsening dyspnea. Her body height was 157 cm and body weight was 48 kg. Blood pressure was 124/97 mmHg and pulse rate was 132 bpm. Chest X-ray presented a cardiothoracic ratio of 0.59 and bilateral lung congestion without interstitial consolidation (Figure 2A). There was no lung consolidation suspected of recurrent lung cancer. Electrocardiograms depicted remaining complete left bundle branch block and poor R progression in V1–4 (Figure 2B). QT duration was 348 msec. Transthoracic echocardiography presented left ventricular end-diastolic diameter of 62 mm and left ventricular ejection fraction of 31%. Plasma B-type natriuretic peptide level was 1175 pg/mL and troponin I level was 58 pg/mL.

**Figure 3:**
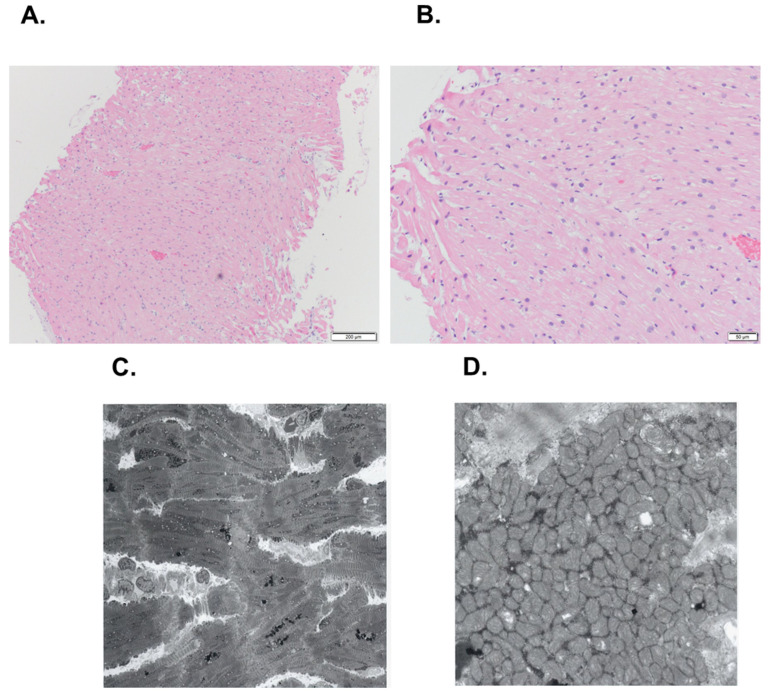
Endomyocardial biopsy obtained from right ventricular septum, displaying mild hypertrophy and size difference in myocardium and mild interstitial fibrosis (hematoxylin and eosin staining, (**A**) ×200, (**B**) ×500). Electronic microscopy of right ventricular septum, demonstrating hypertrophy and variety in size of the myocardium ((**C**) ×700) and small mitochondria ((**D**) ×10,000).

Coronary angiogram proved no coronary artery stenosis. Right heart catheterization revealed a mean right atrial pressure of 9 mmHg, pulmonary artery wedge pressure of 22 mmHg, and a cardiac index of 2.15 L/min/m^2^. Percutaneous end-myocardial biopsy revealed slight myocardial fibrosis and atrophy without infiltration of inflammatory cells (Figure 3A,B). The electron microscopy showed hypertrophy and variety in size of the myocardium (Figure 3C) accompanying smaller mitochondria (Figure 3D). This case presentation was approved by the ethical committee of University of Toyama (R2015154, 11 April 2016). Written informed consent was obtained from the patient.

### 2.3. In-Hospital Course

**Figure 4:**
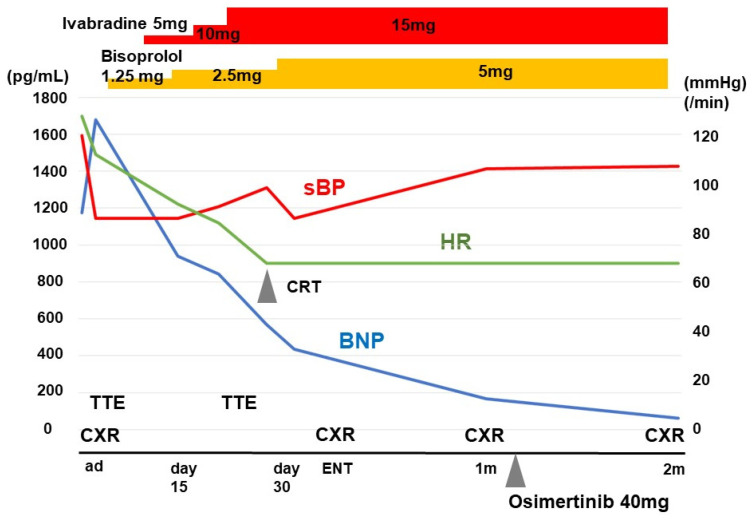
In-hospital course. sBP, systolic blood pressure; HR, heart rate; CRT, cardiac resynchronization therapy; BNP, B-type natriuretic peptide; TTE, transthoracic echocardiography, CXR, chest X-ray.

Osimertinib was terminated (Figure 4). Pulmonary congestion was relatively improved by intravenous administration of diuretics. Heart rate remained around 120 bpm, which was refractory to 2.5 mg of carvedilol. Administration of carvedilol somewhat induced symptomatic hypotension. Termisartan 20 mg was converted to enalapril 1.25 mg. Carvedilol was converted to bisoprolol 1.25 mg. Systolic blood pressure tended to be around 80 mmHg and heart rate around 110 bpm. Further aggressive intervention was required to treat her advanced heart failure.

**Figure 5:**
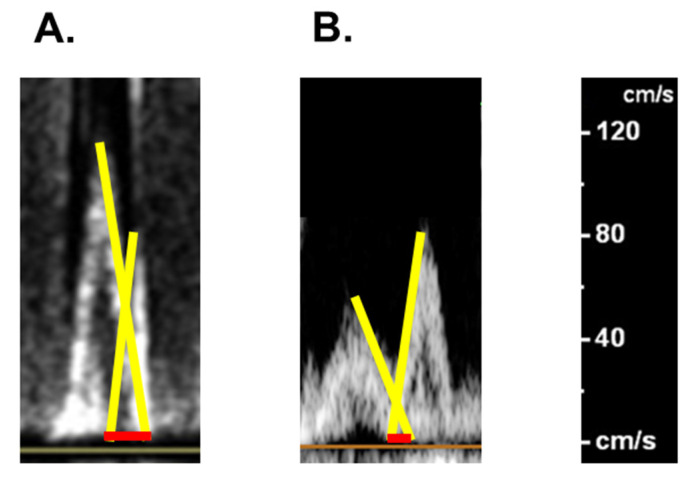
Trans-mitral left ventricular inflows obtained by Doppler echocardiography on admission (**A**) and following the up-titration of ivabradine (**B**). The overlap between the two waves are presented as red bars.

Ivabradine 5.0 mg was initiated to modulate her heart rate. Two left ventricular inflow waves in the trans-mitral Doppler echocardiography obviously overlapped at baseline (Figure 5A). We up-titrated ivabradine dose to minimize the overlap between the two waves, at which cardiac output should be theoretically maximized and optimal reverse remodeling can be expected [4]. Eventually, ivabradine was up-titrated to 15 mg and her heart rate tended to be around 70 bpm with minimized overlap between the two left ventricular inflow waves (Figure 5B).

**Figure 6:**
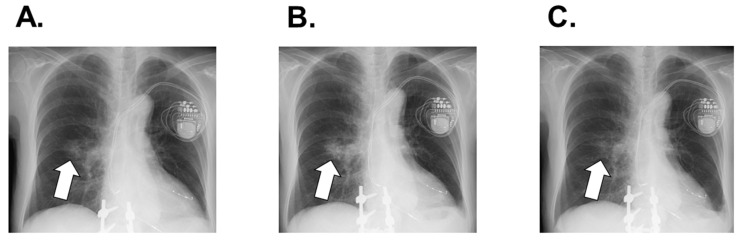
Post-discharge chest X-ray ((**A**), at discharge implying recurrent right lung consolidation; (**B**), 1 month later showing worsening consolidation; (**C**), following 2 months of osimertinib therapy proving remission of consolidation). White arrows show recurrent consolidation.

Her systolic blood pressure increased to around 100 mmHg, which let us up-titrate bisoprolol to 5.0 mg. On in-hospital day 32, transthoracic echocardiography revealed left ventricular end-diastolic diameter of 52 mm, left ventricular ejection fraction of 42%, and plasma B-type natriuretic peptide level of 435 pg/mL. Before discharge, she received implantation of cardiac resynchronization therapy without complications. She was discharged on foot on in-hospital day 33. Chest X-ray at discharge indicated no pulmonary congestion or recurrent right lung consolidation (Figure 6A).

### 2.4. Post-Discharge Course

One month later, transthoracic echocardiography indicated left ventricular end-diastolic diameter of 43 mm and left ventricular ejection fraction of 62%. Plasma B-type natriuretic peptide was 167 pg/mL. Given the worsening of the malignant tumor and relatively controlled heart failure (Figure 6B), osimertinib was initiated again at half dose (i.e., 40 mg). She was followed-up for two months without any comorbidities including heart failure recurrence. Chest X-ray showed regression of right lung consolidation (Figure 6C).

## 3. Discussion

### 3.1. Cardiotoxicity of Osimertinib

Several studies report the cardiotoxicity of osimertinib, although the detailed clinical course of osimertinib-related heart failure remains uncertain [5,6]. A retrospective review of the FDA adverse events reporting system indicated a 2.3% incidence of heart failure during osimertinib therapy [5]. In another Japanese report, 3 patients (2.5%) had heart failure during osimertinib therapy [6].

The detailed mechanism remains unclarified. Unlike direct myocardial injury by anthracycline [2], osimertinib impairs cardiac function via multifactorial mechanisms [7]. EGFR-TKIs might suppress a signal cascade that has a deep association with myocardial homeostasis. EGFR-TKI might suppress the appropriate activity of mitochondria and induce their apoptosis, which was consistent with our spectroscopic findings. EGFR-TKI also suppresses the HER2 receptor and may inhibit appropriate myocardial differentiation.

The reason why only a few patients, including our patient, develop heart failure during osimertinib remains unknown. Given that a previous study reported a decline in mean LVEF in 36 patients receiving osimertinib [6], many patients may have occult worsening of cardiac function during osimertinib therapy.

Our patient did not have most of the known risk factors, including concomitant use of anthracycline, history of thoracic radiation, body mass index above 30 g/m^2^, and baseline low LVEF, except for age over 65 years old and a history of hypertension [7].

### 3.2. Therapeutic Strategy for Osimertinib-Induced Heart Failure

Several therapeutic strategies have been proposed for the general management of cancer therapeutics-related cardiac dysfunction, but the osimertinib-specific strategy remains unestablished.

Screening of potential high-risk patients and careful monitoring using newly proposed echocardiography strain patterns and troponin levels are suggested for early diagnosis and prompt therapeutic intervention [8]. Other indices or artificial intelligence-guided methodologies might be promising for the early detection of cardiac abnormality, including electrocardiographic diastolic index [9]. In our patient, we followed electrocardiography during osimertinib therapy, but we could not prevent her heart failure worsening. Further risk stratification and follow-up methodology specific to osimertinib therapy are warranted given the restricted medical resources.

Given its dose-independency, the termination of osimertinib might be helpful for the recovery of cardiac function. Kunimasa and colleagues reported three patients whose LVEF recovered at least partially by 1–3 months following the termination of osimertinib [6]. Osimertinib was converted to other EGFR-TKIs in two patients and was continued at a half dose in another; no patients had heart failure recurrence. We decided to administer osimertinib again at a half dose, given the worsening of malignant tumor and relatively controlled heart failure owing to the below-discussed optimal medical therapy.

The implication of neurohormonal blockers remains unknown. A systemic review including 14 published articles reported that prophylactic treatment, including neurohormonal blockers, was efficacious for reducing cardiotoxicity in patients receiving chemotherapeutic agents, predominantly anthracyclines [10]. It was challenging to titrate neurohormonal blockers in our patient due to hypotension, which was ameliorated by ivabradine-incorporated heart rate modulation and the maximization of cardiac output targeting minimization of two left ventricular filling waves [11]. The up-titration of neurohormonal blockers in addition to the temporal termination of osimertinib would have facilitated the recovery of cardiac function. 

## 4. Conclusions

In our patient, we believe that the termination of osimertinib alone was insufficient to manage her advanced heart failure. Furthermore, optimal medical therapy was essential to re-start osimertinib for recurrent malignant tumors. Further studies are warranted to validate our proposed strategy.

## Data Availability

Data are available upon reasonable requests.
